# A pilot study using metagenomic sequencing of the sputum microbiome suggests potential bacterial biomarkers for lung cancer

**DOI:** 10.1371/journal.pone.0177062

**Published:** 2017-05-25

**Authors:** Simon J. S. Cameron, Keir E. Lewis, Sharon A. Huws, Matthew J. Hegarty, Paul D. Lewis, Justin A. Pachebat, Luis A. J. Mur

**Affiliations:** 1 Institute of Biological, Environmental and Rural Sciences, Edward Llywd Building, Penglais Campus, Aberystwyth, Ceredigion, United Kingdom; 2 Department of Respiratory Medicine, Prince Phillip Hospital, Hywel Dda University Health Board, Llanelli, United Kingdom; 3 College of Medicine, Swansea University, Swansea, United Kingdom; West Virginia University, UNITED STATES

## Abstract

Lung cancer (LC) is the most prevalent cancer worldwide, and responsible for over 1.3 million deaths each year. Currently, LC has a low five year survival rates relative to other cancers, and thus, novel methods to screen for and diagnose malignancies are necessary to improve patient outcomes. Here, we report on a pilot-sized study to evaluate the potential of the sputum microbiome as a source of non-invasive bacterial biomarkers for lung cancer status and stage. Spontaneous sputum samples were collected from ten patients referred with possible LC, of which four were eventually diagnosed with LC (LC^+^), and six had no LC after one year (LC^-^). Of the seven bacterial species found in all samples, *Streptococcus viridans* was significantly higher in LC^+^ samples. Seven further bacterial species were found only in LC^-^, and 16 were found only in samples from LC^+^. Additional taxonomic differences were identified in regards to significant fold changes between LC^+^ and LC^-^cases, with five species having significantly higher abundances in LC^+^, with *Granulicatella adiacens* showing the highest level of abundance change. Functional differences, evident through significant fold changes, included polyamine metabolism and iron siderophore receptors. *G*. *adiacens* abundance was correlated with six other bacterial species, namely *Enterococcus* sp. 130, *Streptococcus intermedius*, *Escherichia coli*, *S*. *viridans*, *Acinetobacter junii*, and *Streptococcus* sp. 6, in LC^+^ samples only, which could also be related to LC stage. Spontaneous sputum appears to be a viable source of bacterial biomarkers which may have utility as biomarkers for LC status and stage.

## Introduction

Lung cancer is the most prevalent cancer in the world with 1.3 million deaths recorded each year [1]. Lung cancers are classified into various subtypes reflecting their cytology and cellular origins. The main sub-divisions are non-small-cell lung carcinoma (NSCLC) and small-cell lung carcinoma (SCLC). The overall five year survival rate for lung cancer has improved very little over the last 30 years, with only 15% of patients living for five or more years after initial diagnosis [2]. These poor survival rates are primarily due to its late detection, with two thirds of patients diagnosed at a stage where chemotherapy and lung thoracotomy is less likely to be successful [3].

The main risk factor for the development of lung cancer is tobacco smoking, but genetic predisposition also plays a major role [4]; possibly explaining why not all smokers develop the lung condition [5]. A history of previous lung disease such as chronic obstructive pulmonary disease (COPD), chronic bronchitis, tuberculosis and pneumonia has been associated with an increased risk of developing lung cancer [6]. Interestingly, in the “never smokers” group a significantly increased risk of lung cancer was observed only in patients with a previous history of pneumonia and tuberculosis. Such observations suggest that microbial changes–possibly linked to inflammatory events–could be an independent risk factor associated with certain types of risk cancer [7].

Since the link between *Helicobacter pylori* and gastric cancer was identified [8], the possible links between the host and its microbiome, in terms of response, exacerbation or even the initiation of carcinogenesis are receiving increased attention. Changes in the bacterial loads for key species, for example, have been linked to oral squamous carcinoma, colorectal cancer and oesophageal cancer [9]. Within the context of lung cancer, a link between *H*. *pylori* seropositivity and risk of lung cancer has been investigated through the use of serum samples from patients with lung cancer and age-matched controls [10]. Although, no correlation was reported, it did show that a number of people with lung cancer tested seropositive for *H*. *pylori* and there is a possibility it could be present in the lung cancer microbiome. The use of serum in this study highlights how the microbiome-cancer links have been investigated using cancers, such as oral [11–14] and colorectal [15–17] where sampling can be minimally invasive. However, the enclosed nature of the lung complicates sample collection and has involved sampling using bronchoalveolar lavage fluids (BAL), tissue from excised lungs obtained during transplantation surgery [18], or indirectly through serum [10].

In our previous study, we have used sputum to suggest chemical biomarkers linked to lung cancer. Sputum is a complex of mucus, microorganisms, cellular debris and other particles trapped in the lungs by mucus. It provides a non-invasive method of obtaining upper bronchial tract samples that also involves minimal patient discomfort [19]. The production of sputum is a symptom of inflammatory lung airway diseases such as lung cancer, COPD, asthma, and cystic fibrosis, it is often used to provide insight into the underlying malignancies [20]. Indeed, conditions such as asthma [21], COPD [18, 22, 23] and cystic fibrosis [24] have used microbial profiling techniques to reveal potentially important insights into the role that microbes may play in disease aetiology, progression and treatment. Sputum from lung patients has been used to explore, albeit in a culture-dependent method, the microbial flora and the level of antibiotic resistance [25]. A further, culture-independent study using amplicon sequencing suggested that, in sputum, there are significant differences between lung cancer patients and controls, particularly within the *Granulicatella*, *Abiotrophia*, and *Streptococcus* genera [26]. However, to date, there has been no study into the metagenomic composition of the sputum microbiome in lung cancer. Therefore, resolution at the species level of taxonomy has not been possible, and the functional capacity of the microbiome has not been investigated. Other respiratory conditions have been studied with this method, such as cystic fibrosis, though with relatively small sample numbers, such as two [27], five [28], and ten [29].

In this pilot-level study, we aimed to assess the potential clinical usefulness of using the sputum microbiome as a non-invasive sampling medium by which biomarkers for lung cancer status and stage could be obtained. By taking advantage of recent technological advances, that have already been utilised in the human gut microbiome [30] that have reduced both the cost and complexity of metagenomic sequencing, we report on preliminary data that suggests significant taxonomic and functional differences are present in the sputum microbiome of patients with and without lung cancer. Furthermore, we identify the relative abundances of *Granulicatella adiacens*, and six other bacterial species, *Enterococcus* sp. 130, *Streptococcus intermedius*, *Escherichia coli*, *Streptococcus viridans*, *Acinetobacter junii*, and *Streptococcus* sp. 6, as a potential, non-invasive and novel biomarker for lung cancer, and lung cancer progression.

## Methods

### Ethics statement

The MedLung observational study (UKCRN ID 4682) received loco-regional ethical approval from the Hywel Dda Health Board (05/WMW01/75). All procedures undertaken were in accordance with the ethical standards of the Helsinki Declaration (1964 and amended 2008) of the World Medical Association. Written informed consent was obtained from all participants at least 24 hours before sampling, at a previous clinical appointment, and all data was link anonymised before analysis. All methods were carried out in accordance with relevant guidelines and regulations. The sponsor was Hywel Dda University Health Board and neither the funders–Aberystwyth University or NISCHR—nor sponsor had any input into the design or reporting of the study.

### Patient recruitment and sampling

Spontaneous sputum was collected from ten clinical patients, who were referred for further diagnostics at Prince Phillip Hospital, Llanelli, UK, after presentation with lung cancer-like symptoms at their General Practice. Spontaneous sputum samples were taken before bronchoscopic investigation for lung cancer diagnosis. All spontaneous sputum samples were confirmed as sputum, based on bronchial cell content, by a Consultant Pathologist in the Hywel Dda University Health Board Pathology Service.

### Isolation of genomic DNA

Spontaneous sputum samples were transferred, on dry ice, to Aberystwyth University laboratories, were they were thawed on ice for 60 minutes. Subsequently, samples were treated with 5 mL of 30% aqueous methanol and 500 μL of a methanol-dithiothreitol (DTT) solution, made up by adding 2.5 g DTT to 31 mL of 30% aqueous methanol, and then vortex mixed for 15 minutes. Samples then underwent centrifugation at 1500 x g for ten minutes, and the supernatant removed. The remaining pellet was transferred to a PCR grade 1.5 mL microcentrifuge tube. Genomic DNA was extracted from 100 μL of treated sputum using a FastDNA SPIN kit for soil (MP Biomedical, Santa Ana, USA) following manufacturer’s instructions. Bead beating was carried out in a FastPrep-24 machine (MP Biomedical) with three cycles at speed setting 6.0 for 30 seconds, with cooling on ice for 60 seconds between cycles. Genomic DNA was eluted in to 30 μL of DES and dsDNA concentration determined using the Quant-iT dsDNA High Sensitivity assay kit and a Qubit fluorometer (Life Technologies, Paisley, UK). All DNA extractions were completed using the same FastDNA SPIN kit box to minimise the potential effect of extraction kit contamination, as previously reported [31].

### Metagenomic library preparation and sequencing

After extraction of genomic DNA, samples were normalised to 10 ng/μL with PCR grade water (Roche Diagnostics Limited, West Sussex, UK) and 50 ng used to create metagenomic libraries using the Nextera^®^ DNA kit (Invitrogen, San Diego, USA) following manufacturer’s instructions, except that a MinElute PCR purification kit (Qiagen, Ltd Crawley, UK) was used for the clean-up of tagmented DNA. Nextera^®^ DNA libraries were quantified using the Quant-iT dsDNA High Sensitivity assay kit, and approximate library sizes determined by running on a 2% agarose gel alongside HyperLadder IV (Bioline, London, UK). Sample libraries were pooled in equimolar concentrations and sequenced at 2 x 151 bp using an Illumina HiSeq 2500 rapid run, with samples duplicated over two lanes, and following standard manufacturer’s instructions at the IBERS Aberystwyth Translational Genomics Facility.

### Metagenomic sequence analysis

After sequencing, output files for each lane were combined into one file, using the BioLinux 7 environment [32], for each read direction. Sequencing files were uploaded to MG-RAST (v3.2) [33] as FASTQ files. Paired-end reads were joined using the facility available within MG-RAST, with non-overlapping reads retained. Sequences were dereplicated and dynamically trimmed using the default parameters for FASTQ files, and human sequences removed by screening against the *Homo sapiens* (v36) genome, available via NCBI. The MG-RAST pipeline used an automated BLASTX annotation of metagenomic sequencing reads against the SEED non-redundant database [34]. SEED matches can be matched to identity at various taxonomic levels; including genus and species levels. Organism abundances were modelled and exported from MG-RAST using the ‘Best Hit Classification’ after alignment to the M5NR database, with alignment cut-off parameters set at an e-value maximum of 1 x 10^−5^, a minimum identity of 97%, and a minimum alignment of 15. Functional abundances were modelled and exported from MG-RAST using ‘Hierarchical Classification’. SEED matches can also be related to metabolic information, again at different levels of classification. The coarsest level of organization; the generalized cellular function was termed level 1, and the finest, individual subsystems level 3. To normalise for potential variations in sequencing efficacy, sequence abundances were transformed into percentages based upon the total read abundance for each sample at each taxonomic or functional level. Statistical analysis was completed using the MetaboAnalyst 2.0 [35] facility and MINITAB 14 package. Multiple hypotheses testing was not corrected for during statistical analyses. Sequence files can be viewed on MG-RAST via the IDs listed in S1 Table and raw sequence reads, after removal of host DNA, have been deposited at the European Nucleotide Archive under study primary accession number PRJEB9033 and secondary accession number ERP010087.

### 16S rRNA quantitative PCR

Quantitative PCR was completed on neat extracted DNA against standards created through amplification of the 16S rRNA gene of five randomly selected samples (three LC- and two LC+), as previously described [36]. Subsequent qPCR reactions were completed in 25 μL reaction volumes, consisting of 1X SYBR Green Mastermix (Life Technologies), 400 nM of each of the forward and reverse primers, and 1 μL of neat DNA extract, with the reaction volume being made up with PCR grade water (Roche Diagnostics Limited, West Sussex, UK). Reactions were run using a C100 thermal cycler (BioRad, Hercules, USA) and CFX96 optical detector (BioRad), with data captured using CFX Manager software (BioRad), under conditions of 95°C for 10 minutes, 40 cycles of 95°C for 15 seconds and 60°C for 60 seconds, followed by a melt curve consisting of a temperature gradient of 60°C to 95°C in 0.5°C increments, each for 5 seconds.

## Results

**Fig 1:**
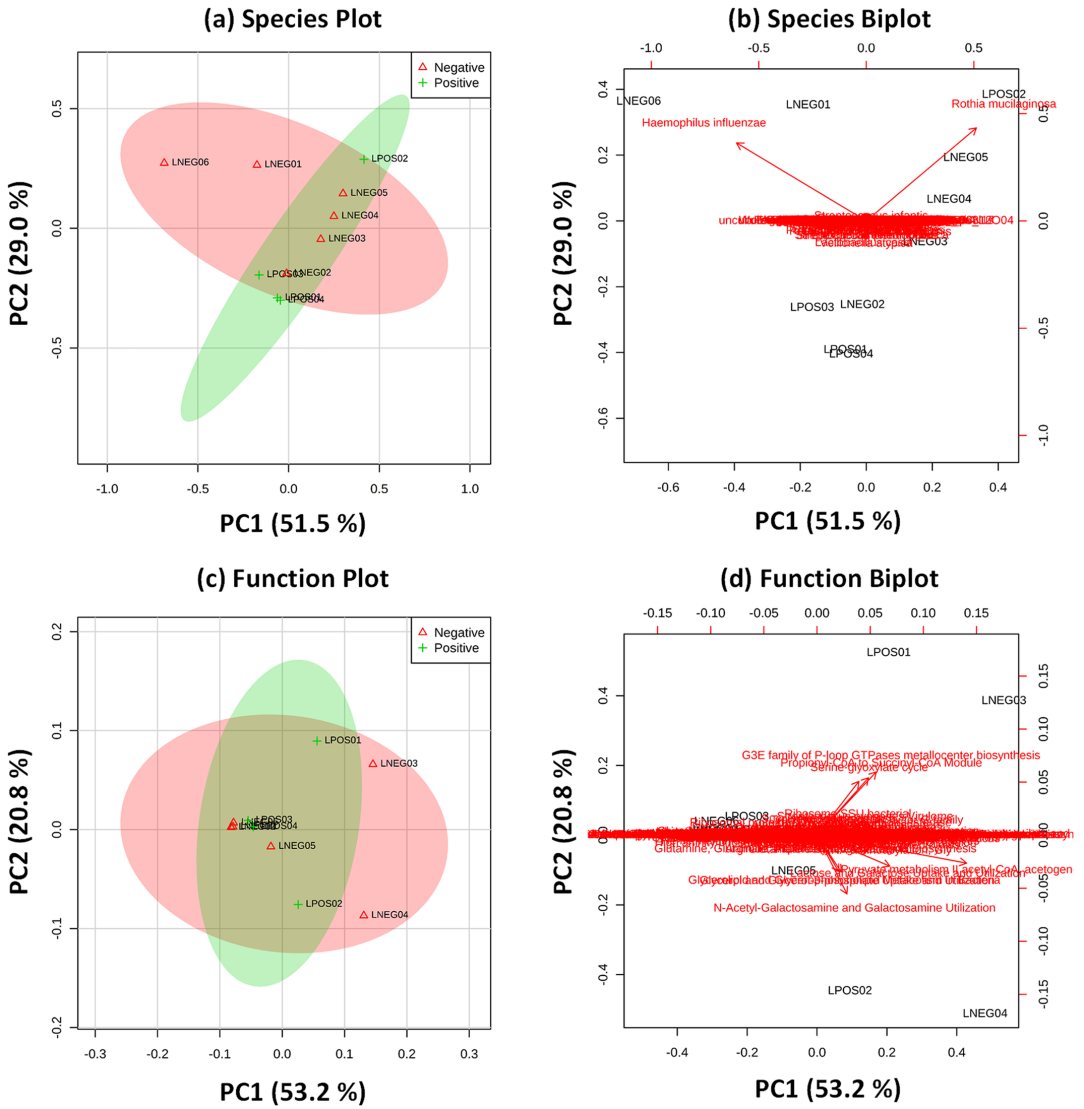
Alpha diversity group means. Alpha diversity measures of species richness, as calculated by the MG-RAST analysis pipeline, show no significant difference between either positive or negative lung cancer groups. This suggests that any changes to the lung microbiome as a result of a malignancy are not large-scale community shifts.

After histological investigation of the ten patients referred with lung cancer-like symptoms, four patients were diagnosed with lung cancer (one squamous cell NSCLC, one adenocarcinoma NSCLC, one large cell carcinoma NSCLC, and one where a bronchoscopy was not possible and a radiological diagnosis was required), and six were found to be negative for lung cancer presence. Summarised patient information is shown in Table 1, and full individual patient information in S1 Table, with no discernible differences between the two patient groups being observed. Of particular importance, no significant (*P* = 0.197) differences were observed between smoking pack years of either LC group. DNA extractions for LC+ groups were a mean of 83.28 ng/μL and for LC- were 91.42 ng/μL, with no significant (*P* = 0.786) differences evident between groups. Sequencing statistics, both pre and post and quality control process are summarised in S2 Table, alongside corresponding one-way ANOVA *P* values. In all but one of the sequencing statistics, “identified rRNA features”, no significant differences were present, suggesting that the HiSeq 2500 sequencing platform and subsequent bioinformatic analysis using MG-RAST did not introduce any level of bias which may affect results interpretation. Additionally, no significant difference was observed between the bacterial loads of the two groups, based on estimated 16S rRNA copy number (*P* value = 0.616), data not shown, nor between the alpha diversity measures of species richness between samples, 1 (*P* value = 0.778). (Fig 1).

**Table 1:** Average patient characteristics for negative and positive lung cancer groups. Data are means for either the negative or the positive lung cancer groups. Standard deviations, where appropriate, are given in brackets. FEV_1_% of predicted is forced expulsion volume of lungs in one second, as a percentage of the predicted value for that patient. CO level is carbon monoxide in parts per million concentration. *P* Value column indicates value from one-way ANOVA analysis.

	LC-	LC+	*P* Value
**Number**	6	4	N/A
**Age**	58.8 (14.8)	73.3 (7.2)	0.112
**Gender**			
**Male**	4	2	N/A
**Female**	2	2	N/A
**Smoking Status**			
**Current**	4	3	N/A
**Ex**	2	0	N/A
**Never**	0	1	N/A
**Smoking Pack Years**	57.7 (35.4)	28.8 (24.6)	0.197
**Infection Present**			
**Yes**	0	0	N/A
**No**	6	4	N/A
**Antibiotic Use**			
**Yes**	1	0	N/A
**No**	5	4	N/A
**CO Level (ppm)**	21.0 (24.5)	7.3 (8.0)	0.317
**FEV1% of Predicted**	77.1 (22.3)	69.3 (16.0)	0.559

**Fig 2:**
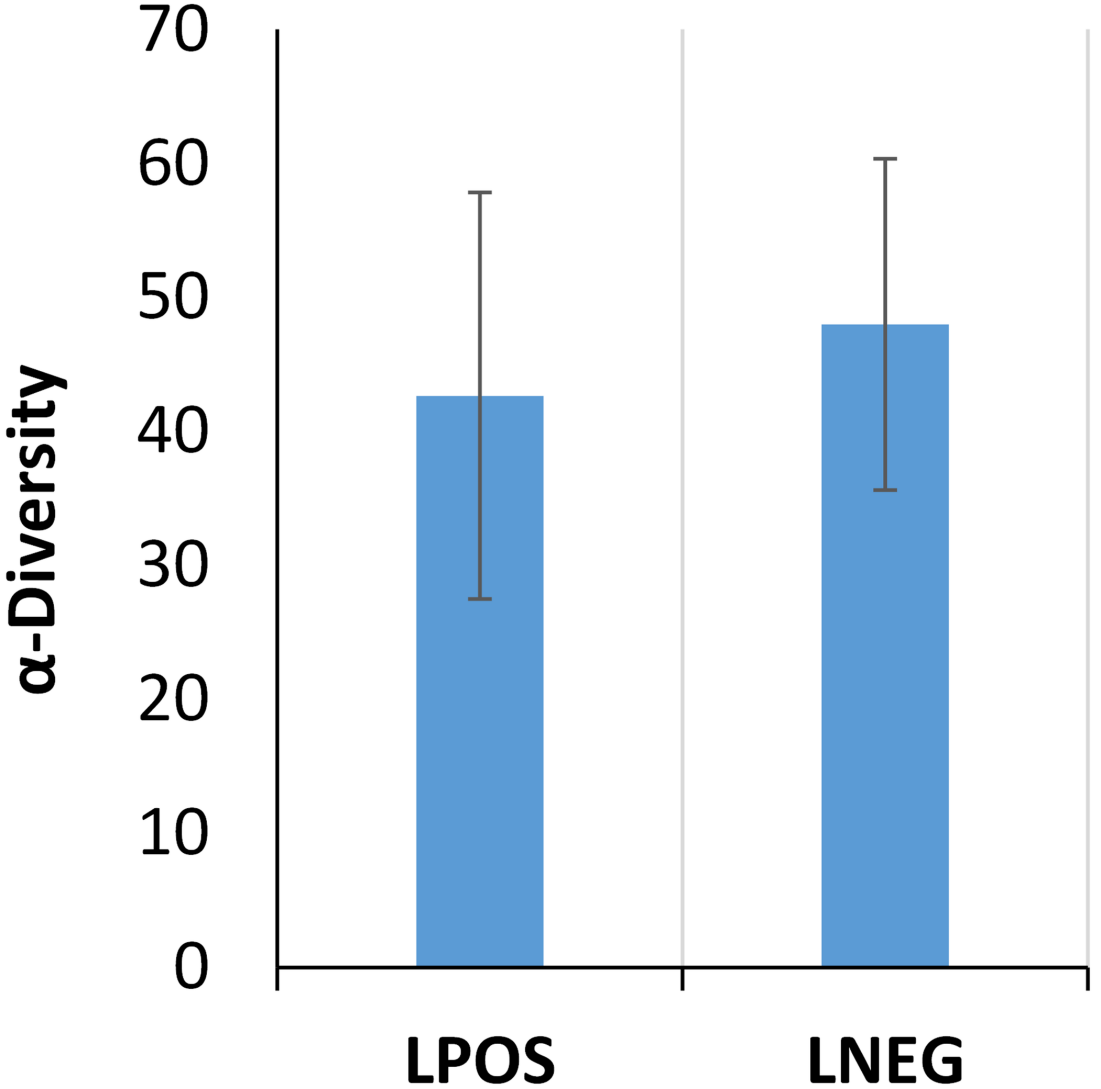
Principal component analysis of taxonomic and functional classifications. PCA plots and biplots, to identify factors leading to observed groupings, created using the MetaboAnalyst platform using normalised percentage abundance of (A and B) bacterial species and (C and D) level 3 functional alignments. LC- samples are indicated by red symbols and LC+ by green symbols. Coloured areas indicate 95% confidence intervals of PCA groupings as calculated by MetaboAnalyst. For biplots, red-letter annotations show the factor contributing to the observed separation.

At the species level of taxonomy (Fig 2A), and at level 3 of functional classifications (Fig 2C), principal component analysis, created using normalised bacterial species abundance was suggestive that the presence of a malignancy within the lungs does not change either the taxonomic composition or the functional capacity of the sputum microbiome on a substantial scale. However, more subtle changes in individual bacterial species or functional classifications may be evident which could have utility as disease biomarkers.

As metagenomic sequencing is able to resolve to the species level of taxonomy, the ‘core’ microbiome of both negative and positive lung cancer patients was investigated (Table 2), as this is likely to give greater insight into the microbiome than a genus level profile. A total of seven species were found to be present in all ten samples, with *Streptococcus viridans* found to be significantly (*P* = 0.042) higher in the positive lung cancer samples. Six further species were found to be present in all of the LC^-^ but not all of the LC^+^ samples, but due to their variation within the LC^-^ group were significantly different in their level of abundance. However, a total of 16 bacterial species were found in all of the positive lung cancer samples, but not all of the LC^-^ samples, with *Granulicatella adiacens* (*P* = 0.015), *Streptococcus intermedius* (*P* = 0.023), and *Mycobacterium tuberculosis* (*P* = 0.036) significantly higher in the LC^+^ group.

**Table 2:** Average percentage abundance of species present in ‘core’ microbiome. Average percentage abundance of species present in the negative and positive lung cancer groups, with corresponding *P* values from one-way ANOVA. % column shows average abundance, St. Dev. column shows standard deviation, and Count column shows the number of patients in each group in which the species was found, out of the total number of patients. The top division represents species present in all samples, the second division those found in all negative samples, and the bottom division those found in all positive samples.

Species	LC-			LC+			*P* Value
	%	St. Dev.	Count	%	St. Dev.	Count	
**Found in all samples**							
*Streptococcus viridans*	0.02%	0.01%	6 / 6	0.10%	0.04%	4 / 4	**0.042**
*Streptococcus thermophilus*	1.73%	1.33%	6 / 6	5.96%	2.51%	4 / 4	0.115
*Ochrobactrum anthropi*	0.48%	0.20%	6 / 6	0.95%	0.32%	4 / 4	0.188
*Streptococcus pneumoniae*	7.30%	1.84%	6 / 6	15.17%	6.72%	4 / 4	0.200
*Enterococcus faecalis*	0.10%	0.03%	6 / 6	0.25%	0.13%	4 / 4	0.203
*Salmonella enterica*	0.01%	<0.01%	6 / 6	0.05%	0.04%	4 / 4	0.271
*Neisseria meningitidis*	1.04%	0.73%	6 / 6	0.79%	0.30%	4 / 4	0.756
**Found in all LC- samples**							
*Escherichia coli*	0.01%	0.01%	6 / 6	0.06%	0.04%	3 / 4	0.207
*Fusobacterium nucleatum*	0.16%	0.14%	6 / 6	<0.01%	<0.01%	2 / 4	0.310
*Haemophilus influenzae*	22.29%	17.38%	6 / 6	4.30%	3.70%	3 / 4	0.346
*Streptococcus parasanguinis*	9.87%	4.24%	6 / 6	4.75%	4.75%	1 / 4	0.398
*Streptococcus pyogenes*	0.24%	0.11%	6 / 6	0.31%	0.12%	3 / 4	0.659
*Veillonella parvula*	1.60%	0.64%	6 / 6	2.01%	1.84%	2 / 4	0.805
**Found in all LC+ samples**							
*Granulicatella adiacens*	<0.01%	<0.01%	1 / 6	0.07%	0.03%	4 / 4	**0.015**
*Streptococcus intermedius*	<0.01%	<0.01%	2 / 6	0.06%	0.02%	4 / 4	**0.023**
*Mycobacterium tuberculosis*	<0.01%	<0.01%	5 / 6	0.01%	<0.01%	4 / 4	**0.036**
*Enterococcus sp*. *130*	0.01%	0.01%	5 / 6	0.06%	0.02%	4 / 4	0.050
*Streptococcus sp*. *6*	0.01%	0.01%	5 / 6	0.06%	0.02%	4 / 4	0.050
*Acinetobacter junii*	0.01%	<0.01%	5 / 6	0.03%	0.01%	4 / 4	0.112
*Streptococcus salivarius*	0.14%	0.10%	4 / 6	3.23%	2.46%	4 / 4	0.195
*Shewanella sp*.	<0.01%	<0.01%	3 / 6	0.01%	<0.01%	4 / 4	0.206
*Streptococcus vestibularis*	0.01%	0.01%	5 / 6	1.31%	0.74%	4 / 4	0.208
*Lactobacillus paracasei*	<0.01%	<0.01%	1 / 6	2.61%	2.60%	4 / 4	0.234
*uncultured bacterium*	<0.01%	<0.01%	4 / 6	<0.01%	<0.01%	4 / 4	0.266
*Lactococcus lactis*	0.02%	0.01%	4 / 6	0.02%	0.01%	4 / 4	0.379
*Neisseria gonorrhoeae*	0.29%	0.20%	5 / 6	0.17%	0.08%	4 / 4	0.500
*Rhodococcus erythropolis*	0.09%	0.11%	3 / 6	0.13%	0.06%	4 / 4	0.628
*Stenotrophomonas maltophilia*	0.10%	0.05%	5 / 6	0.20%	0.08%	4 / 4	0.662
*Staphylococcus aureus*	0.42%	0.49%	5 / 6	0.20%	0.14%	4 / 4	0.666

**Fig 3:**
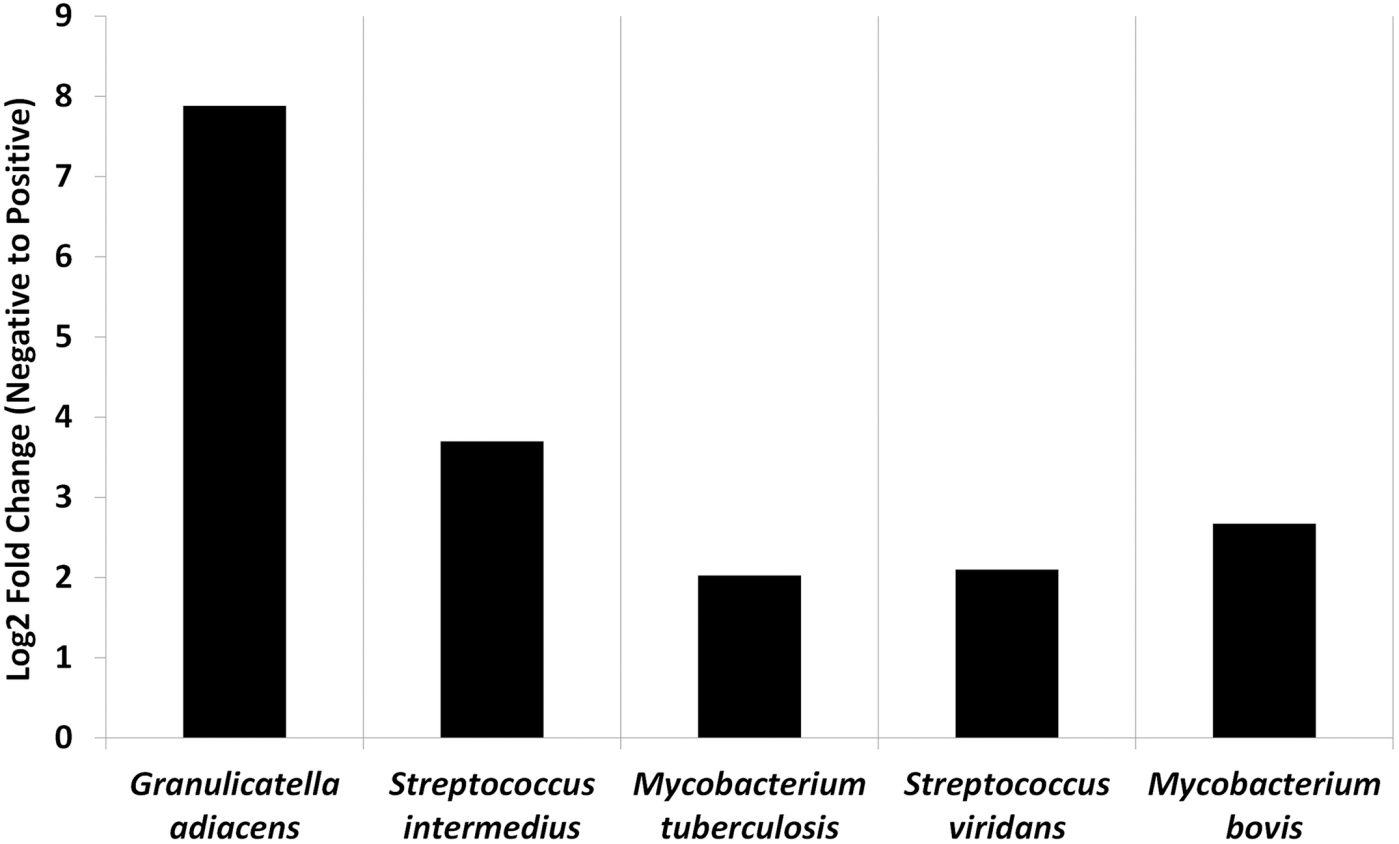
Significant fold changes in species abundance from negative to positive for lung cancer. Using the online features of MetaboAnalyst 2.0, significant fold changes, as determined by t-Tests with *P* values <0.05, were identified. Five species, from three genera, were all higher in positive lung cancer samples, with *Granulicatella adiacens* and *Streptococcus intermedius* showing the highest change.

Additionally, at the taxonomic level, significant (*t*-Test *P* < 0.05) fold changes in regards to species abundance between positive and negative lung cancer cases were identified (Fig 3). This reflected differences in the ‘core’ microbiome changes shown in Table 2, namely significantly higher abundances of *G*. *adiacens*, *S*. *intermedius*, and *M*. *tuberculosis*, in positive cases, with additional significant increases evident in the abundance of *Streptococcus viridans* and *Mycobacterium bovis*.

**Fig 4:**
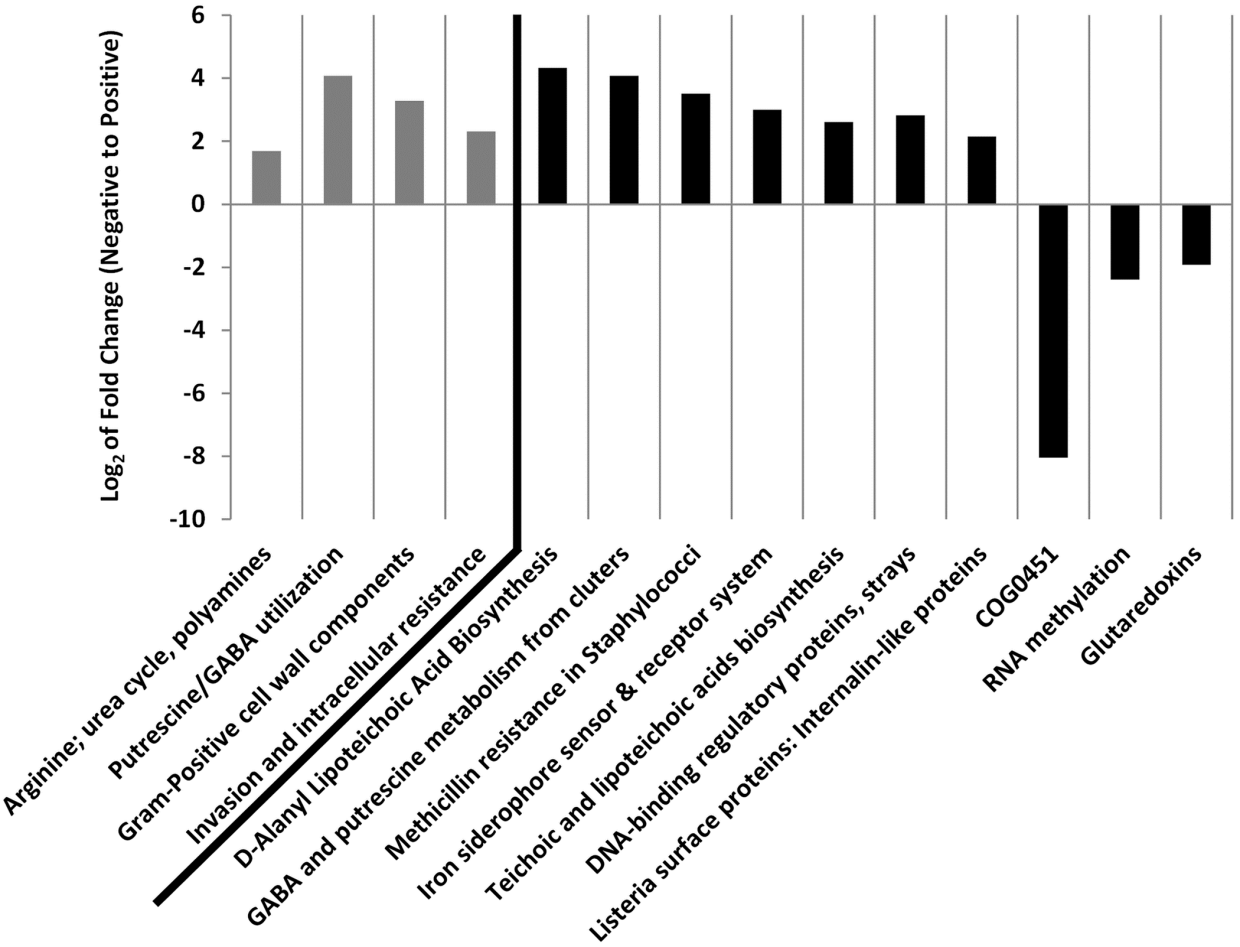
Significant fold changes in levels 2 and 3 functions from negative to positive lung cancer. Using MetaboAnalyst 2.0, significant fold changes of Level 2 (grey bars) and 3 (black bars) functional alignments, as determined through t-Tests with *P* values <0.05, were identified. A total of four Level 2 functional alignments were higher in positive lung cancer, alongside seven Level 3 functions. Three Level 3 functional alignments were lower in positive lung cancer samples.

Significant fold changes in functional alignments were also identified. At the crudest level of functional classification, Level 1, no differences were evident. However, at Levels 2 and 3 (Fig 4), significant differences were observed. At Level 2, four functional classifications were higher in positive lung cancer samples. At Level 3, seven functional classifications were higher in positive lung cancer samples, whilst three were lower, when compared to negative lung cancer samples. These differences appeared to be across a wide range of biological functions.

**Fig 5:**
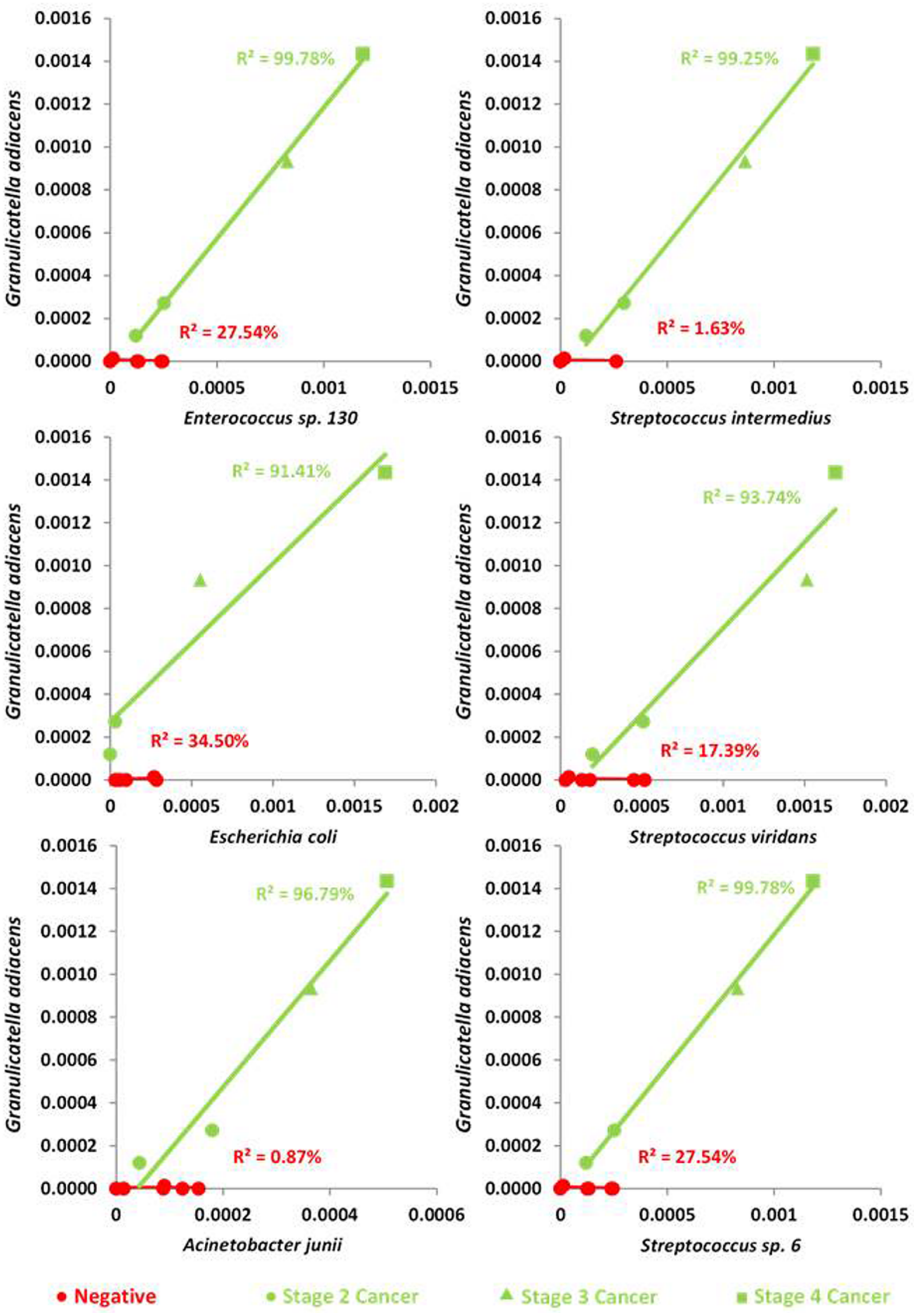
Regression analysis suggests importance of *G*. *adiacens* in positive lung cancer samples. Species regression analyses were completed, and those with an R^2^ value of greater than 80% were plotted to identify differing relationships between negative and positive lung cancers. This type of relationship was shown to exist between *G*. *adiacens* and six other species, with a strong positive relationship present in positive lung cancer samples, and no correlation evident within negative lung cancer samples. Normalised percentage abundances are shown on *x* and *y* axes.

To evaluate the potential of using metagenomics to identify novel biomarkers for lung cancer and lung cancer progression, both a species level (S3 Table) and Level 3 functional regression analyses (S4 Table) were completed. Those regressions with an R^2^ value of 80% (chosen as an arbitrary cut-off; data not shown for regressions with R^2^ below 80%) or more were plotted to identify those with differing relationships between negative and positive lung cancer groups. From this method of analysis, *G*. *adiacens* was identified as having positive correlations (*P* value of regression relationship less than 0.001 in all instances) with six other bacterial species (Fig 5) in positive lung cancer samples, but not in negative lung cancer samples. Additionally, when LC^+^ cancer stages were plotted against targeted bacterial abundance, a pattern with disease progression was observable. Although suggestive that relative abundances of some bacterial species could indicate lung cancer progression, this will require confirmation with a larger cohort of patient samples.

## Discussion

The role of the microbiome in a range of respiratory conditions has been well documented; however, lung cancer has received only minimal attention. The lung cancer microbiome has been detailed, at the genus level, in female non-smokers from Xuanwei, China, through the use of amplicon sequencing. Interestingly, significant differences were only detected between sputum samples, and not buccal samples, suggesting a localised effect in the bronchial tree of the lung [26]. This study suggested a potential role of household coal burning exposure, and its effect on the lung microbiome in patients with lung cancer, rather than tobacco smoking, which is the most common cause of lung cancer in more economically developed countries [37]. In this pilot-level study, we looked to address this, and to develop a more in-depth view of the microbiome with clinically relevant samples.

Through the use of metagenomic sequencing, we have identified a number of bacterial species that are increased in abundance in patients with lung cancer, than in those without. Furthermore, we have also identified *G*. *adiacens* as having a significant positive relationship with six other bacterial species, *Enterococcus* sp. 130, *Streptococcus intermedius*, *Escherichia coli*, *Streptococcus viridans*, *Acinetobacter junii*, and *Streptococcus* sp. 6. This significant correlation is only observed in patients positive for lung cancer. The *Granulicatella* genus has been identified as being significantly higher in the sputum of non-smoking lung cancer cases [26], suggesting that it may be a true reflection of lung cancer state, rather than a by-product of tobacco smoking. The *Granulicatella* genus, and *G*. *adiacens* specifically, is a difficult organism to culture, which may be the limiting factor that explains the minimal study that has been conducted into it [38]. It has however, been associated with endocarditis [39] and septicaemia [40]. The sputum microbiome is an understudied area within respiratory microbiology. This may be surprising given that the production of sputum is typically a symptom of lung dysfunction but may reflect the fact that comparison to ‘healthy’ samples is difficult. This stated, many of the bacterial genera and species that we identified in the LC- patients have also been reported in previous studies investigating changes in the sputum microbiome associated with lung disease [26, 41]. This suggests that our observations are robust and represent an accurate reflection of the bacterial composition of the lung microbiome.

The changes in *G*. *adiacens*, as a commensal bacterium and an opportunistic human pathogen, seem likely reflect a change in an “ecological” niche, such as in sputum composition [42], within the lungs of patients with lung cancer. Of the six other bacterial species associated with *G*. *adiacens* in LC^+^ patients, none appear to have been previously linked to lung cancer in the literature. They may also be responding to a changes in the cancerous lung, or potentially, exist in a synergistic relationship with *G*. *adiacens* which enables their higher abundance. Regardless of the biological basis for these significantly higher abundances seen, they nevertheless have the potential to act as biomarkers for lung cancer, in regards to both lung cancer status, but also in staging, due to the pattern of abundances seen in Fig 5. Clearly, given the small scale nature of this pilot study, albeit large for metagenomic studies, these findings should only be taken as suggestive. For example, it is possible that false positives are reported as a result of multiple hypotheses testing in the correlation analyses. Furthermore, due to the limited study size, we were unable to separate our patient cohort into individual test and validation cohorts–which is the ‘gold’ standard for biomarker discovery studies. We were also limited in the statistical tests that could be performed such as controlling for multiple hypothesis testing and non-parametric analysis. These points stated, it is highly relevant that our findings were still in line with previously reported studies identifying members of the *Granulicatella* genus as biomarkers for lung cancer status, thereby implying the veracity of our results. Validation of the bacterial species identified in this study, primarily *G*. *adiacens*, as potential biomarkers of lung cancer status and stage must be completed in larger cohorts which possess sufficient statistical power to allow for true sensitivity and specificity rates to be calculated.

Crucially, metagenomic sequencing allows the field of microbiomics to move beyond characterising the microbiome simply in terms of its taxonomic composition, and more towards understanding how its functional capability shifts in response to disease state. Here we have found a total of four Level 2 classifications that are significantly higher in patients positive for lung cancer, including those involved in arginine use, urea cycle, putrescine and gamma-aminobutyric acid (GABA) utilisation, and invasion and intracellular resistance. Interestingly, elevated levels of polyamines, such as putrescine and GABA, been associated with a range of cancers including lung malignancies [43]. Polyamines offer a rich nitrogen source of bacteria, and elevated levels associated with lung cancer could explain why there are significantly more associated alignments in positive lung cancer cases. At Level 3 of functional classification, seven functions were significantly higher, and three significantly lower, in the positive lung cancers. Some of these increases reflected Level 2 changes, but others, such as higher levels of iron siderophore sensors and receptor system alignments, further suggest that changes in the cancerous lung are reflected by changes in the lung microbiome. Elevated iron levels are associated with lung cancer [44], and as iron is essential for many cellular functions in bacteria, it is not unexpected that elevated levels of iron associated with lung cancer would result in a selective pressure to reflect this in the microbiome. Functional changes are not unexpected in the lung microbiome as a response to malignancy formation, but this study is the first to confirm that such synergy exists. This suggests that a systems biology approach towards studying the lung microbiome is required to better understand this relationship.

An emerging issue within microbiome research is that of contaminated DNA extraction kit and reagents which has the potential to impact both 16S rRNA amplicon and shotgun metagenomic studies. This appears to be of substantial importance when assessing microbial communities of low biomass [31]. Within this body of work, no significant differences in terms of DNA extraction concentrations were observed between LC status groups. Furthermore, the DNA extraction concentration suggests that the sputum microbiome is not one with a low biomass, and thus unlikely to be affected by issues of contaminated DNA extraction kits and other reagents.

Our use of spontaneous sputum is a well-established diagnostic medium for lung cancer because of its non-invasive collection, and because it is symptomatic of lung cancer as a disease. Therefore, it offers a viable alternative to radiography based diagnoses for high-throughput, non-invasive, and low-risk screens [45]. However, it should be appreciated that sputum production is localised to the upper bronchial tract, and particularly the bronchial tree. As microbiome studies in other respiratory diseases have shown, including in COPD [18] and cystic fibrosis [24], spatial differences can exist within the lungs and therefore, sputum should only be taken as representative of the microbiome in the upper bronchial tract. Furthermore, the microbiome present within sputum samples is likely to consist of a number of taxa present within the oral cavity, which may be present at orders of magnitude greater than those taxa with are present within the lower respiratory tract. Although this may impair the potential insight into the lung microbiome gained through the use of sputum, it does not exclude it as a viable sample medium to be used in screening for lung cancer.

## Conclusions

This novel pilot-level study has expanded upon our knowledge of the microbiome in patients with lung cancer, using clinically relevant control samples, particularly in regards to the functional capacity of the microbiome, and its taxonomic composition at the species level. Additionally, we have demonstrated the strength of using metagenomics to identify potential biomarkers for disease state and progression, namely *G*. *adiacens* and its correlations in abundance with a range of bacterial species, which could have clinical use. However, due to the small sample number in this pilot study, more work is needed to confirm these suggestive relationships, and whether they are observable in earlier stage lung cancers, and whether they are able to differentiate between different LC histology.

## Supporting information

S1 Table Individual patient details, medical and drug histories, and cancer histology. Full patient information for negative and positive lung cancer patients, showing age, gender, smoking history and clinical history for all patients, with lung cancer histology for lung cancer positive patients.
(XLSX)

S2 Table Sequencing statistics for negative and positive lung cancer groups. Average read statistics for pre- and post-quality control (QC), for each group, alongside one-way ANOVA *P* values. Analysis shows no significant differences in all bar one, identified rRNA features, suggesting that the sequencing approach, and subsequent analysis using the MG-RAST pipeline, used in this study has not introduced any discernible bias between the two groups. Predicted protein/rRNA features are hypothetical features contained within reference databases used by MG-RAST.
(XLSX)

S3 Table Table of regression values between all species identified in both sample groups. Table of regression values identified between all species identified in both sample groups, with those pairings with a R^2^ value greater than 0.8 highlighted in green.
(XLSX)

S4 Table Table of regression values between all level 3 functional alignments identified. Table of regression values identified between all Level 3 functional alignments identified in both sample groups, with those pairings with a R^2^ value greater than 0.8 highlighted in green.
(XLSX)
